# Do psychological factors affect outcomes in musculoskeletal shoulder disorders? A systematic review

**DOI:** 10.1186/s12891-021-04359-6

**Published:** 2021-06-19

**Authors:** Ali Sheikhzadeh, Maria M. Wertli, Shira Schecter Weiner, Eva Rasmussen-Barr, Sherri Weiser

**Affiliations:** 1 Department of Orthopedic Surgery, Occupational and Industrial Orthopedic Center (OIOC), NYU Langone Orthopedic Hospital, 63 Downing Street, New York, NY 10014 USA; 2 Graduate Program in Ergonomics and Biomechanics (ERBI), Graduate School of Arts and Sciences, New York University, New York, USA; 3 Department of General Internal Medicine, Bern University Hospital, University of Bern, Bern, Switzerland; 4 Horten Centre for Patient Oriented Research and Knowledge Transfer, University Hospital Zurich, Pestalozzistrasse, Zurich, Switzerland; 5 School of Health Sciences, Touro College, New York, NY USA; 6 Department of Neurobiology, Karolinska Institutet, Care Sciences and Society, Division of Physiotherapy, Huddinge, Sweden

**Keywords:** Conservative intervention, Surgical intervention, Modifiable psychological factors, Treatment outcome, Predictors

## Abstract

**Background:**

Psychological factors may impact recovery in patients undergoing treatment for shoulder complaints. The aim of this review is to systematically analyse the evidence for the effect of modifiable psychological factors (MPF) on outcome, for patients with musculoskeletal shoulder disorders undergoing conservative or surgical treatment. MPF refers to factors that may change with intervention.

**Methods:**

This is a systematic literature review. Five databases searched (MEDLINE, CINAHL, Cochrane Library, Embase and PsycInfo), for longitudinal studies investigating the influence of MPF on prognosis of patients with shoulder disorders, all diagnoses, undergoing clinical interventions (conservative or surgical). Level of evidence was determined using Scottish Intercollegiate Guidelines Network (SIGN) methodology. Moderate and high quality evidence was included. We extracted all MPF, categorized constructs into the following domains: beliefs (self-efficacy, expectation of recovery), coping (catastrophizing, avoidant coping), and affect (depression, anxiety). We evaluated constructs for its predictive value of at least one outcome. Outcomes were informed by this review. Evidence was classified into three categories: evidence for, inconclusive evidence, and evidence against.

**Results:**

Of 1170 references, 40 distinct publications based on 35 datasets were included (intervention type: 20 surgical; 20 conservative). Overall, 22 studies (20 cohort studies and 2 RCTs) were classified as high quality and 18 studies (16 cohort studies, 2 RCTs) were classified as moderate quality. Outcomes reported included pain, disability/function, perceived recovery, physical and mental health, and work status. Based on the review, of the psychological constructs explored, these data would suggest that expectation of recovery, catastrophizing, avoidant coping, depression, and anxiety may predict outcome for patients managed surgically. In patients undergoing conservative intervention the evidence was either against (catastrophizing, depression, anxiety) or inconclusive (self-efficacy, expectation of recovery, avoidant coping) for the predictive value of psychological factors on outcome.

**Conclusions:**

Five constructs were predictive of outcome for surgically managed patients. This suggests that implementing the biopsychosocial approach (i.e., preoperative screening, intervention by a trained clinician) may be advantageous for patients recommended for shoulder surgery,,. The same is not indicated for conservatively managed patients as no conclusive association of MPF with outcomes was noted. The importance of other MPF on outcome requires further investigation.

## Introduction

### Background

Shoulder conditions are the third most common musculoskeletal complaint [1, 2]. Only 50 % of patients with a new episode of shoulder pain experience complete recovery within 6 months and pain persists in 40% for more than 1 year [3]. In those who seek care, there is limited understanding of how to identify patients who may or may not respond to interventions [4]. Therefore, we need to understand barriers to and facilitators of recovery in patients with shoulder pain.

To improve treatment outcomes for shoulder complaints, modifiable factors that influence the prognosis should be identified. The focus of this review is on psychological factors. Modifiable psychological factors (MPF) are patient cognitions and emotions associated with health conditions that may impact recovery, and may respond to treatment [4, 5]. Exploring the relationship between MPF and outcome is valuable, as effective management may improve outcomes [6, 7]. MPF are different than psychological traits and refractory psychiatric diagnoses that are more difficult to manage, such as bipolar disorder and pervasive depressive disorder, and not considered in this review. Some MPF have been recognized as impacting recovery in other musculoskeletal conditions [1, 2, 8–11]. Maladaptive pain beliefs, negative affective reactions and poor coping are indicators of psychological distress that may influence both the short and long-term outcomes of treatments in patients with spine, hip and knee conditions [5, 12–14].. Conversely, self-efficacy and positive expectation of recovery are coping resources that have been associated with better functional outcomes in patients with musculoskeletal disorders [10, 11]. Kendall and Burton propose that in the absence of red flags suggestive of an emergent medical situation, all musculoskeletal conditions that limit activity may be treated like low back pain [15]. This treatment would include advice for self-care, education on expectation of a good recovery and instruction to continue with usual activity as tolerated. Despite compelling evidence to monitor and address MPF in patients with spine pain as part of routine care evidence to monitor and address MPF in patients with spine pain as part of routine clinical care [16, 17], to date there is equivocal evidence to support the importance of MPF in MSD [11, 18–22]. As such, these factors typically are not part of routine clinical evaluation and treatment for patients with MSD [23, 24].

Recent reviews explored psychological factors in various patient groups, including those receiving conservative and surgical care [18, 25], conservatively managed patients only [19, 20], patients with selective diagnoses, [21, 25–28], patients undergoing arthroplasty [29] or with conditions associated with chronic shoulder pain [11, 25]. The heterogeneity of these diagnoses makes it difficult to compare the conclusions. In addition, methodologic limitations and variability of previous reviews was also noted [11, 18, 22, 29]. Therefore, [11, 25] the current reviews provide a limited perspective on the relationship between MPF and outcomes in patients with musculoskeletal shoulder disorders (MSD).

The aim of this literature review was to systematically summarize the current evidence on the importance of MPF on outcome in patients receiving care (conservative or surgical) for MSD. The MPF that may be found to be associated with outcome in MSD includes patient beliefs, coping and affect. Unlike previous systematic reviews that focused on some MPF and did not subcategorize studies based on intervention, our aim was to capture studies on all MPF in surgical and conservative studies to better identify those that predict outcomes. This review included all phases of shoulder disorders (acute, subacute, chronic) and all MPF referenced in the reviewed studies, to gain insights regarding the relationship between MPF and MSD.

## Methods

This systematic review followed the recommendation of the Preferred Reporting Items for Systematic Reviews and Meta-analyses (PRISMA) statement [30].

### Search strategy

The framework to determine the research questions, search strategy and criteria for inclusion was defined by the authors by consulting the relevant literature on MPF. We searched five databases, without any language and date range limits, in September 2019: MEDLINE (EBSCOhost), CINAHL (EBSCOhost), Cochrane Library, Embase (Elsevier), and PsycInfo (EBSCOhost), seeking literature for all psychological factors found to be associated with shoulder pain and disability/function, and focused on those considered to be modifiable [31]. An updated search was conducted in December 2020.

The search was conducted with the help of a research librarian (MG). Two detailed search strategies are depicted in Appendix 1.

To ensure the completeness of the literature search, one reviewer (MW) conducted an electronic hand search of the four most often-retrieved journals and added all potentially eligible references not retrieved by the systematic search. In addition, two reviewers (MW, EB) examined bibliographies of included studies and review articles related to the research question, and relevant references were considered for full-text review (inclusion and exclusion criteria applied). We further searched clinical trials.gov for additional trials relevant to the topic and searched the grey literature after consulting with experts in the field. In potentially relevant studies with insufficient details for data extraction, we contacted the study authors for additional information.

### Inclusion and exclusion criteria

Included were all longitudinal studies (cohort studies, randomized controlled trials (RCT), and studies on registry data) investigating patients with shoulder complaints undergoing conservative or surgical treatment for the shoulder disorder. Studies were eligible when they included the influence of MPF on the prognosis or treatment outcome. Excluded were experimental studies (i.e., identification of genetic markers) in which clinical interventions were not used to modify outcome (i.e., pain, function), cross-sectional studies, case series, epidemiological studies, and studies on patients younger than 18 years of age. Studies of personality traits and psychiatric conditions were excluded. Although we did not specifically exclude studies on joint arthroplasty, the search was not set up to identify all studies on total shoulder joint replacement. Therefore, excluded studies on joint arthroplasty for the current review.

### Data collection and abstraction

Two reviewers (MW and ERB), a physician and a physical therapist with extensive clinical and research experience, screened all references independently by title and abstract. Disagreements were discussed and resolved by consensus or by third-party arbitration (SSW), a physical therapist. For any study where questions arose regarding psychological constructs or outcome measures, a psychologist (SW, co-author) was consulted. References with insufficient information in the title or abstract to assess eligibility, were included in the full text review. All full texts were then appraised by both reviewers independently (MW and ERB) for inclusion or exclusion. Alternative researchers with specific language proficiencies were used for non-English language references, with no language restrictions. In the case of several publications for the same cohort without change in outcome or follow-up duration, the most recent publication was chosen and missing information from the previous publication was added. Systematized criteria were defined to extract specific variables from each reference and were followed by each reviewer. All information needed to describe the study population and methodology was collected: study setting, study design, number of patients, age, proportion of women, intervention, and follow-up duration. In addition, the methods of assessment and information on the type of analysis of the prognostic, predictive or mediating factors were extracted. The inclusion/exclusion criteria guided this process.

### Assessment of study quality

A quality rating was assigned based on the risk of bias, using the Scottish Intercollegiate Guidelines Network (SIGN) methodology checklist for cohort studies and randomized clinical trials and the overall quality was rated as high, moderate, or low [32]. The ratings were as follows: high quality (++), most (≥60%) of the criteria fulfilled; moderate quality (+), some criteria fulfilled (< 60%); and low quality (−), few or no criteria fulfilled. Two reviewers (MW and EBR) assessed each reference. Any discrepancies were resolved by another member of the research team (SSW). High and moderate quality studies were included in this review.

### Definition of terms

For this study, MPF are defined as those factors that may be expected to change with appropriate therapeutic intervention and are therefore states rather than traits. We utilized a framework of psychological domains [16] and modifiable constructs extracted from the included studies (Table 1) in order to synthesize the findings. It is important to note that there is no gold standard for the definition and classification of MPF. Therefore, for those constructs that may fall into more than one domain, we sought the guidance of a clinical mental health expert to inform the distinct classification based on the context in which the constructs were considered in the studies. This allowed for the classification of all constructs within one domain.

**Table 1:** Definitions of domains and constructs

Domains and definitions	Constructs and definitions
**Beliefs**: cognitive responses to pain	● **Self-efficacy**: belief in one’s ability to be successful at a task ● **Expectation of recovery**: belief that one will return to the premorbid state
**Coping**: active or palliative responses to pain	● **Catastrophizing**: thoughts that something is much worse than it is ● **Avoidant coping**: unhelpful avoidance of dealing with a stressful situation
**Affect**: emotional response to pain	● **Depression**: feelings of extreme sadness ● **Anxiety**: worrisome or fearful thoughts

The term prognostic factor is used to describe a MPF that influences or predicts the course or outcome of a shoulder disorder. The prognostic value of a psychological factor is based on the reported results and conclusions of the primary studies. No predefined outcomes were identified for this review. Study outcome was extracted from each included reference based on the reported measure of assessment.

We classified studies based on patients’ duration of pain as subacute (< 12 weeks), chronic (> 12 weeks) or a mixed duration of shoulder complaints.

### Classification of evidence

All included studies were grouped based on the MPF addressed, time from onset and clinical intervention (conservative, surgical). We evaluated each construct based on the number of studies that reported it as a predictor of at least one outcome or not a predictor of any outcome. Outcomes were purposefully not predefined, as our objective was to identify all outcomes that have been included in studies on MPF in patients with MSD. If the number of studies with results showing that a construct was predictive of outcome was greater than the number of studies showing it was not predictive, we considered the construct predictive. If the opposite was true, then we considered the construct to not be a predictor of outcome. In those cases where an equal number of studies found evidence for and against the predictive value of the construct, the evidence was found inconclusive. Based on these criteria, the evidence was classified into three categories: Category 1) Evidence for – a majority of the studies found the construct to be a predictor of outcome; Category 2) Inconclusive evidence – An equal number of studies found evidence for and against the predictive value of the construct, Category 3) Evidence against-a majority of studies did not find the construct to be a predictor of outcome.

## Results

### Study selection

**Fig. 1:**
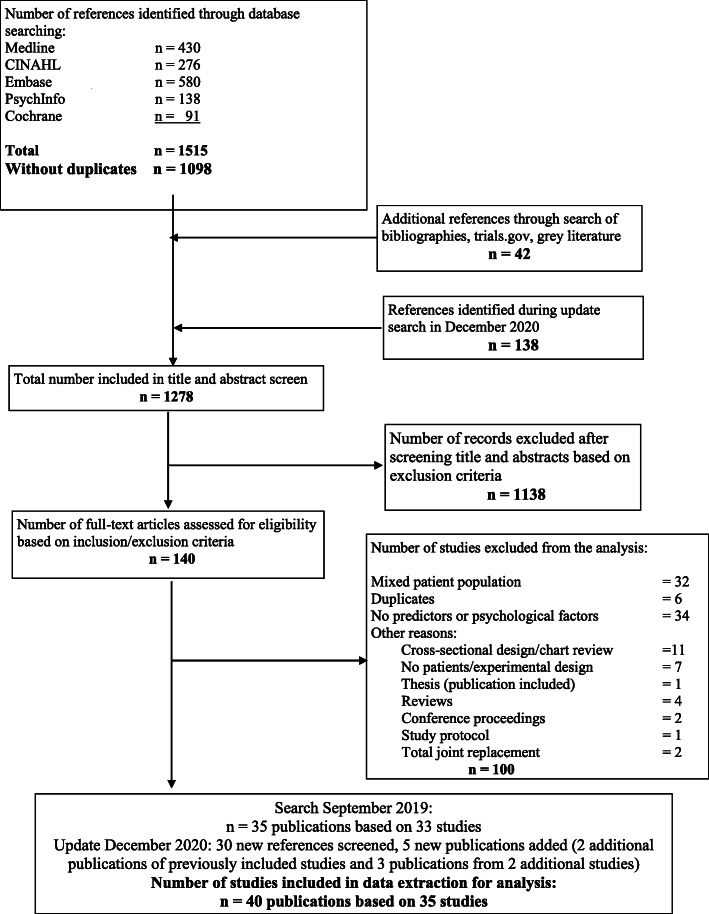
Systematic review flowchart

In the initial search 1140 references were screened, and 121 full-text articles assessed for eligibility. After excluding 86 publications, 35 publications based on 33 patient data sets were included for data extraction and analysis, hereafter referred to as 35 studies. The main reasons for exclusion were mixed patient populations without reporting specific results for subjects with shoulder complains (*n* = 31) and studies that did not assess MPF (*n* = 26, Fig. 1). In the updated search conducted on December 20, 2020 we identified 138 additional references. After title and abstract screen, an additional 19 references were read in full text. Finally, we included 5 additional publications (2 additional publications of previously included studies and 3 publications from 2 additional studies). In total, the narrative analysis reflects our review of 40 distinct publications based on 35 patient data sets, hereafter referred to as 40 studies.

### Baseline characteristics

Of the 40 included studies, four were randomized clinical trials. There were 20 studies on conservative intervention and 20 on surgical intervention. Follow-up duration ranged from end-of-treatment to 12 months. The studies represented a broad spectrum of shoulder diagnoses, representative of a typical clinical population (Table 2).

**Table 2:** Characteristics of the reviewed studies. Bold font indicates high quality studies based on the SIGN review

Author, year	Design	^a^Setting	Diagnosis	SS n	N (% Female^b^)	Age: mean	Intervention	Treatment duration	Follow-up	Drop-out: %	Outcome variables
**Conservative treatment**											
Berk et al. 1977 [33]	RCT	Advertisement recruitment, USA	Shoulder pain due to tendonitis or bursitis	NR	42 (28%)	47	Group 1) Acupuncture - positive milieu, Group 2) Acupuncture - negative milieu, Group 3) Placebo acupuncture - positive milieu, Group 4) Placebo acupuncture - negative milieu	All groups 4 sessions	All groups 1 week after the end of treatment	NR	Subjective pain (VAS)
**Chester et al, 2016, Chester et al 2019** [4, 34]	Prosp. Cohort	PT clinic England	Shoulder or arm pain aggravated by shoulder movement	1000	1030 (56%)	57	Non-specified PT treatment reflecting usual care	NR	6 weeks and 6 months after initiating PT treatment	25%	Pain and disability (SPADI, QuickDASH)
**Ekeberg et al. 2010** [35]	RCT, secondary analysis	Outpatient PT and rehabilitation department, Norway	Patients with a clinical diagnosis of rotator cuff disease included in the RCT	NR	104 (61%)	52	Group 1 systemic corticosteroid injection (gluteal region), group 2 ultrasound guided corticosteroid injection.	1 injection	6 weeks	2%	Pain and disability (SPADI) Global assessment score
**Engebretsen et al. 2010** [36]	RCT, secondary analysis	Physical Medicine and Rehabilitation clinic, Norway	Chronic subacromial pain	NR	104 (50%)	48	Group 1) Supervised exercise, Group 2) Radial extracorporeal shockwave therapy	Group 1). twice a week for maximum of 12 weeks, Group 2) once a week for 4–6 weeks.	12 months	14%	Pain and disability (SPADI) and work status
**Engebretsen et al. 2020** [37]	Prosp. Cohort	Physical Medicine and Rehabilitation clinic, Norway	Shoulder pain lasting for ≥6 weeks (2015–2016).	NR	167 (55%)	46	Usual care	Not specified	6 months	29%	Main symptoms, disability (SPADI), work status
Geraets et al. 2005 ^16^ [38]	RCT	GP clinic and advertisement; Netherlands	Chronic shoulder complaints	132	176 (55%)	52.2	Group 1) Graded exercise; Group 2) Usual GP care	Group 1) Up to 18 sessions over 12 weeks. Group 2) PRN	12 weeks	Group 1) 9%; Group 2) 21%	Performance of daily activities (Patient report, and SDQ)
**Karel et al, 2017** [39]	Prosp. Cohort	PT, Netherlands	New episode of shoulder pain	360	389	49.9	PT, not specified	NR	6.5 Months	30%	Global perceived effect scale, Pain (NRS), Disability (SPADI)
**Kennedy et al. 2006** [40]	Prosp. Cohort	PTs center, Canada	PTs included 5 clients undergoing treatment for soft tissue shoulder complaints	NR	361 (54%)	50	PT treatment	NR	12 weeks or end of treatment	NR	Disability (DASH): response patterns
Kromer et al. 2014 ^24^ [41]	RCT, secondary analysis	PT clinic, Germany	Subacromial pain	90 (45 per group)	90 (51%)	51.8	Group 1) Exercise; Group 2) Exercise, manual therapy shoulder and cervical spine, and education	Both groups 10 treatments in 5 weeks followed by 7 weeks home exercise	3 months	2%	Pain and disability (SPADI)
Kuijpers et al. 2006 ^25^ [42]	Prosp. Cohort	GP clinic, Netherlands	Acute shoulder pain	NR	587 (50%)	51	Usual care including medical management and physical therapy	Not defined	6 weeks and 6 months	8%	Patient perceived recovery
**Kvalvaag et al. 2018** [43]	Double blind RCT	Department of Physical Medicine and Rehabilitation, Norway	Subacromial pain syndrome lasting at least three months	For RCT *n* = 143	143	47	Radial Extracorporeal Shock Wave Therapy (rESWT) + supervised exercises vs. sham rESWT + supervised exercises	Once per week for 4 weeks	12 months	9%	Pain and disability (SPADI), work status
**Laslett et al. 2015** [44]	Prosp. Cohort	Primary care/ PT clinic, New Zealand	Acute shoulder pain	NR	161 (49%)	44	Clinical exam, shoulder x-ray, diagnostic anaesthetic injection in bursa + AC-joint or intra-articular glenohumeral joint, after 3 weeks usual PT care	NR	3 weeks, 3, 6, and 12 months	38%	Pain and disability (SPADI)
**O’Malley et al. 2004** [45]	Prosp. Cohort	Orthopedic clinic, USA	Shoulder pain	NR	199 (47%)	52	Various interventions	NR	3 months	39%	Function (FLEX-SF)
**Reilingh et al. 2008** [46]	Prosp. Cohort	GP, Netherlands	Shoulder pain	NR	587 (50%)	51	Various interventions	NR	6 months	8%	Pain (NRS)
**Ryall et al. 2007** [47]	Prosp. Cohort	Primary care and PT clinics, U.K.	Shoulder pain	NR	222 (of 375 with arm pain)	NR	Various interventions	NR	12 months	17% (of total population)	Subjective pain report
Sindhu et al. 2012 [48]	Retro. analysis of prosp.collected data	Outpatient rehab clinics, various locations throughout the United States	Shoulder impairment	NR	3362 (54%)	54	Conservative care	NR	End of treatment	53%	Shoulder function (CAT)
**Smedbråten et al.** **2018** [49]	Registry study	Outpatient Physiotherapy Norway, FysioPol database	Shoulder impairment	NR	145 (72%)	44	Exercises physiotherapy	5 weeks (IQR 3 to 6)	End of treatment	NA	Pain (NRS) Function (PSFS)
**Van der Windt et al. 2007** [50]	Prosp. Cohort	Primary care clinic, Netherlands	Acute shoulder pain	NR	344 (48%)	51	Usual care by GP (Group 1), including steroid injection if indicated (Group 2)	NR	3 Months	12%	Perceived recovery (VAS), Disability (SDQ)
**Wolfensberger et al. 2016** [51]	Retro. Study	Rehabilitation clinic, Switzerland	Chronic nonspecific shoulder pain, on work disability	NR	287 (18%)	47	Interdisciplinary care	4–5 weeks, at least 2 to 3 h of daily (excl. weekend)	End of treatment	49%	Disability (DASH), Pre-post change of pain (Patient Global Impression of Change)
**Surgical treatment**											
Cho et al. 2015 [52]	Prosp. Cohort	Tertiary care institution, Korea	Rotator cuff tear	40	58 (57%)	57	Rotator cuff repair	NA	3, 6, 12 months post-surgery	19%	Pain (VAS), Shoulder Pain and function (UCLA, ASES)
Dambreville et al. 2007 [53]	Prosp. Cohort	Orthopedic surgical department, France	Patients undergoing surgery for shoulder complaints	NR	86 (36%)	48	Several procedures (ablation of calcification, rotator cuff repair, arthroplasty)	NA	1 month	NR	Pain (VAS))
**Dekker et al. 2016** [54]	Retro. analysis of prospectively collected data	Orthopedic surgical department, UK.	Subacromial impingement	NR	61 (NR)	54	Arthroscopic subacromial decompression	NA	6 Months	28%	Pain (VAS) function and pain (OSS)
George et al. 2008 [55]	Prosp. Cohort	Orthopaedics Sports Medicine Institute, USA	Patients scheduled for shoulder arthroscopy, nonspecific diagnosis	NR	58 (41%)	50	Shoulder arthroscopy	NA	3–5 months post-surgery	19%	Pain (BPI)
**George et al. 2015, George et al. 2016, Simon et al. 2020** [56–58]	Prosp. Cohort	Orthopaedics Sports Medicine, USA	Patients scheduled for shoulder arthroscopy, nonspecific diagnosis	360	150 (34%)	43	Shoulder arthroscopy	NA	12 months	NR	George et al. 2015: Pain (BPI), George et al. 2016: Pain (BPI), Disability (Quick-DASH)
Henn et al. 2007 [59]	Retro. analysis of prospectively collected data	Department of Orthopaedic Surgery, USA	Primary repair of a chronic rotator cuff tear	NR	12 (42%)	56	Three rotator cuff repair techniques: open repair, mini open repair, arthroscopic repair.	NA	12 months	NR	Pain (VAS) Function (DASH, SST, Quality of life (SF-36)
**Jain et al. 2018** [60]	Prosp. Cohort	Sports/Shoulder clinics in 3 academic and 1 community setting, USA	Symptomatic (≥4 weeks) rotator cuff tears scheduled for surgery	NR	50 (38%)	59	Surgery rotator cuff tear	NA	3, 6, 12, 18 months		Pain and disability (SPADI)
**Koorevaar et al. 2016** Koorevaar et al. 2018 [61, 62]	Prosp. Cohort	Single center teaching hospital, Netherlands	Patients eligible for shoulder surgery	NR	315 patients (2016),142 (2018) (44%)	54	Surgery shoulder	NA	After treatment (2016) and 12 months (2018)	Postoperative 9%, 12 months 22%	Disability DASH; MCID Anchor based (global rating for function and pain)
Lau et al. 2019 and Lau et al. 2020 [63, 64]	Retro. analysis of prosp. collected data	Single surgical unite, single surgeon, USA	Surgery for rotator cuff repair (01/2011–06/2017) and ≥ 1-year follow-up. Excluded were previous surgery, arthritis, fracture.	NR	187 (34%)	59	Arthroscopic rotator cuff repair in a chair position. All surgeries were performed by one surgeon.	NA	Mean 47.5 months	Complete case analysis	Disability (ASES), pain, quality of life (WORC)
Oh et al. 2012 [65]	Prosp. Cohort	Single center, all surgeries performed by the first author	Patients undergoing surgery for rotator cuff disorders, failed 3 months of conservative management	NR	128 (45%)	59	Arthroscopy-assisted mini open repair or arthroscopic repair	NR	≥12 months	NA	Simple Shoulder Test (SST), Constant-Murley, SF-36 physical function
Potter et al. 2015 [66]	Prosp. Cohort		Patients aged ≥18 years, scheduled for shoulder arthroscopy for shoulder pain secondary to a reparable full-thickness rotator cuff tear.	NR	70 (26%)	61	Patients underwent arthroscopic rotator cuff repair with one of three surgeons (PEG, RTB, RZT) between October 2011 and December 2013	NA	12 months	NA	Pain (VAS), Simple Shoulder Test (SST), ASES
**Ravindra et al 2018** [67]	Prosp Cohort	Single orthopedic department, USA	Patient scheduled for arthroscopic rotator cuff repair with confirmed (MRI) partial or full rotator cuff tear	NR	93 46%	56	Arthroscopic subacromial decompression, acromioplasty, labral debridement, distal clavicle excision, and biceps tenotomy or tenodesis as indicated	NA	Post-surgery 12 months	21.5%	VAS pain scores ASES
Thorpe et al. 2018 [68]	Prosp Cohort	Surgery performed by 6 surgeons in 1 private & 2 public hospitals, Australia	Patients scheduled for shoulder surgery for partial or full rotator cuff tear	NR	124 (37%)	54	Surgery for rotator cuff repair with or without subacromial decompression (*n* = 55) and arthroscopic subacromial decompression only (*n* = 43)	NA	3, 12 months	10%	Pain and function sub- scores (ASES)
Valencia et al. 2014 [69]	Prosp. Cohort	Orthopaedic Sports Medicine Institute, USA	Patients scheduled for shoulder arthroscopy nonspecific diagnosis	NR	78 (28%)	47	Shoulder arthroscopic surgery	NA	3 and 6 months	6%	Pain (BPI), shoulder disability (DASH)
Woollard et al. 2017 [70]	Prosp. Cohort	University Clinic, Sports Medicine, USA	Patients scheduled for arthroscopic subacromial decompression	Yes, 50 pat. 80% power	62 (63%)	46	Arthroscopic subacromial decompression with /without supraspinatus repair	NA	6 months after surgery	25%	Function: (WORC and DASH) Global Rating of change
Yeoman et al. 2012 [71]	Prosp. Cohort	Department of Orthopaedics Surgery, Scotland	Patients scheduled for shoulder arthroscopy	49	31 (67%)	55	Shoulder arthroscopic surgery	NA	6 weeks	0	Shoulder pain and function (OSS), Pain (VAS)

^a^ Setting: represents location of intervention; *SS calc* sample size calculation; ^b^ Female: percentage reported or author estimate; *RCT* randomized controlled trial, *NR* not reported, *NA* not applicable

*4DSQ* Four-Dimensional Symptom Questionnaire, *ASES* the American Shoulder and Elbow Surgeons’ Scale, *BPI* Brief Pain Inventory, *CBT* cognitive behavioural therapy approach, *DASH (and quickDASH)* (Quick) Disability of the Arm, Shoulder and Hand Questionnaire, *EQ-5D* EuroQol- 5 Dimension, *FABQ* Fear Avoidance Beliefs Questionnaire (FABQ-P: physical activity subscale; FABQ-W, work subscale), *FLEX-SF* Flexilevel Scale of Shoulder Function, *GE* graded exercise, *HADS* Hospital Anxiety and Depression Scale, *HSCL-25* Hopkins Symptoms Checklist, *MCID* A minimal clinically important difference, *MODEMS* Musculoskeletal Outcomes Data Evaluation and Management System, *NPRS* Numeric Pain Rating Scale, *NRS* Numeric Rating Scale, *OSS* Oxford Shoulder Score, *PCCL* Pain Coping and Cognition List, *PCS* Pain Catastrophizing Scale, *PSFS* Patient Specific Functional Scale, *PT* physical therapy, *RCT* randomized controlled trial, *SDQ* Shoulder Disability Questionnaire, *SF-36* Short Form Survey, *SPADI* Shoulder Pain and Disability Index, *SST* Simple Shoulder Test, *TSK* Tampa Scale of Kinesiophobia, *UC* usual care, *UCLA Scale* The University of California at Los Angeles Shoulder Score, *VAS* Visual Analog Scale, *WORC* Western Ontario Rotator Cuff Index

### Study quality

Risk of bias in 40 studies was assessed using the SIGN method (Appendix 2A). In all tables, high-quality studies included in this manuscript (Appendix 2A) are indicated by bold typeface. Twenty cohort studies were rated as high quality and 16 studies rated as moderate quality. Two randomized clinical trials were rated as high quality and two were rated as moderate. Overall, 20 (50%) of included studies were rated as high quality, 14 studies related to conservative care, and 6 studies related to surgical intervention. Most studies did not provide a formal sample size calculation. Six (30%) of the conservative studies reported a required sample size and met the requirement. Five (25%) surgical studies reported a required sample size; three studies met the required sample size, and two studies did not (150 instead of 360 patients, same data set for both studies).

### Study outcomes and measures

Various outcomes were noted in the reviewed literature and included those related to pain, disability/function, perceived recovery, physical and mental health, and work status. The most common outcomes noted in the reviewed literature were pain (16 (40%) publications), disability/function (21 (58%) publications), combined pain and disability/function (19 (48%) publications). Outcome measures most commonly utilized in the reviewed studies included the Visual Analog Scale (VAS) for pain (8 (23%) publications), the Disabilities of the Arm, Shoulder and Hand (DASH and QuickDASH) measuring function (8 (20%) publications), and the Shoulder Pain and Disability Index (SPADI) (12 (30%) publications). All outcome measures are listed in Tables 3 and 4.

**Table 3:** Predictive utility of psychological factors on the outcome after conservative treatment for shoulder complaints. Bold font indicates high quality studies. Bold font indicates high quality studies based on the SIGN review

Authors	Quality	Outcome (measure)	Beliefs		Cognitive Style		Affect: Distress		Effect
			1.Self efficacy / Coping	2. Expectation of recovery	3. Catastrophizing	4. Avoidance Coping Style	5. Depression	6. Anxiety / Worry/ Fear	
Berk et al. 1977 [33]	(+)	Pain (VAS)		**–**					Acupuncture in a negative and a positive milieu resulted in similar pain reduction (*p* = 0.053).
**Chester et al. 2016** [4]	(++)	Pain and disability (SPADI, QuickDASH)					**–**	**–**	Patient expectation of ‘complete recovery’ compared to a ‘slight improvement’ as ‘a result of physiotherapy treatment’ (Beta 12.43, 95% CI 8.2–16.67 for 6 months). Depression and anxiety: no consistent association in the multivariate models.
**Chester et al. 2019** [34]	(++)	Pain and disability (SPADI, QuickDASH)	**+**	**+**					Additional analysis using the risk groups as Chester et al. 2016. Using Classification and Regression Tree (CART) analysis, the authors categorized the subjects into three groups (based on the predictor analysis Chester 2016) – those with high, moderate or low levels of pain and disability. There was a positive association. Between pain and disability at baseline and at follow-up. Those with high pain and disability and high self-efficacy scores (PSEQ≥48) were less likely to have continued high levels of pain. Patients with moderate levels of baseline pain and disability and high expectation of recovery had better outcomes at 6 months than those with low expectations of recovery. Patients with low baseline pain/disability and low pain self-efficacy (PSEQ < 41) had increased likelihood of persistent pain. No external validation of the CART.
**Ekeberg et al. 2010** [35]		Pain and disability (SPADI) Shoulder complaint (^a^ADI)		**-** **-**			**-** **-**		Distress (HSCL-25) and self-efficiency for pain (single item question) not associated with SPADI and shoulder complaint as measured by Global Assessment Score at 6 weeks.
**Engebretsen et al. 2010** [36]	(++)	Pain and disability (SPADI) Work status (^a^ADI)	**-** **-**				**-** **-**		Self-efficacy was significant in the univariate analysis but not in the final model for disability and not significant for return to work. Distress (Hopkins Symptoms Checklist) was not significant in the univariate analysis.
**Engebretsen et al. 2020** [37]	(++)	Pain and disability (SPADI)						**+**	TSK predicted outcome SPADI scores at 6 months based on multivariate regression (Beta 0.76, 95% CI 0.27–1.2, *p* = 0.003). HSCL-10 did not predict pain and disability outcomes, however high baseline scores were associated with high HSCL follow up scores. Örebro screening questionnaire predicted sick-leave at follow-up: OR 1.075 (95% CI (1.03 to 1.12)), *p* = 0.001 however no specific psychological factors were extracted in the analysis.
Geraets et al. 2005 [38]	(+)	Shoulder disability (SDQ)					**+**	**–**	Coping measured by PCCL, and FABQ and TSK measured but not used in the model; DSQ N.S.; Significant relationship between reduction of severity of main complaint (graded exercise group vs usual Care group Beta 7.6, 0.9–14.3) at 12 weeks and pain reduction (26.8, 95% CI 19.3–34.4) and baseline depression scores (8.3, CI 0.1–16.6, 4DSQ); Anxiety (4DSQ) N.S.
Karel et al., 2017 [39]	(++)	Perceived recovery (^a^ADI)					**–**		No significant association between psychological factors and perceived recovery: OR for patient reported “no anxiety/depression” in (EQ-5D) 1.8 (95% CI 0.9–3.6), *p* = 0.06. Note: on the anxiety/depression dimension of the EQ-5D, only one patient scored “very anxious/depressed”, 83% reported “not anxious/depressed”, and 16% reported “moderately anxious/depressed”.
Kennedy et al. 2006 [40]	(++)	Pain and disability (SPADI)					**+**		Four patterns of response were found: cluster A had high disability at baseline and less improvement over a long course; cluster B had high disability at baseline but had a quick, steep improvement course; cluster C had moderate disability at baseline and, like A, a slow course with less improvement; and finally cluster D with lower disability at baseline and a short swift change to very low disability. Clusters C and D had a higher baseline Mental Component Score (SF-36 MCS, higher score indicates better health) than clusters A & B. In the final model, one unit increase on the MCS is associated with approximately a 1.1 increase in the odds ratio of being in clusters C and D vs. clusters A and B. Therefore, a 10-unit increase on the MCS would be associated with approximately a 2.6 increase in the odds ratio of being in clusters C and D vs. clusters A and B.
Kromer et al. 2014 [41]	(+)	Pain and disability (SPADI)			**–**	**–**			Catastrophizing, measured by PCS, did not influence the baseline disability and change score in disability at 3 months; FABQ-P contributed significantly to baseline disability but not to the change score in disability at 3 months.
Kuijpers et al. 2006 [42]	(+)	Perceived recovery (^a^ADI)			**–**	**–**	**–**	**–**	Coping with pain (PCCL) N.S.; FABQ and TSK N.S. Univariate analysis for pain at 6 weeks but not for 6 months (4DSQ) significant, but not in the multivariate model; In univariate analysis for pain at 6 weeks but not for 6 months (4DSQ), not in the multivariate model; Anxiety (4DSQ) significant in univariate analysis for pain at 6 weeks but not for 6 months, not in the multivariate model.
**Kvalvaag et al. 2018** [43]	(++)	Pain and disability (SPADI) Work status (^a^ADI)		**+** **-**			**-** **-**		Univariate significant: SPADI baseline score, age, gender, work status, marital status, education, duration of pain, medication, self-efficacy for pain, outcome expectations, general health status, number of PT sessions and emotional distress. Multivariate: low patient expectations were the strongest predictor of a negative outcome (Beta −4.2, 95% CI −7.2 to −1.1, *p* < 0.01). Self-efficacy, distress (HSCL-25) were no longer significant. Outcome expectation, self-efficacy, distress univariate not significant.
**Laslett et al. 2015** [44]	(++)	Pain and disability (SPADI)				+		–	Six months follow-up FABQ, OR 1.03 (95% CI 1.00–1.07), and 12 months FABQ OR 1.01 (95% CI 1.03–1.17) in the multivariate analysis. SF-8 lower SF mental score in the multivariate model OR 0.93 (95% CI 0.85–1.01) 3 weeks, not significantly associated with outcome (3, 6, 12 months follow-up) in the univariate analysis.
**O’Malley et al. 2004** [45]	(++)	Function (FLEX-SF)		**+**					In the final statistical model, patients with higher outcome expectancies (Patient Shoulder Expectancy Fulfilment measure) reported better 3-month shoulder functioning (Beta 0.46, *p* = 0.002).
**Reilingh et al. 2008** [46]	(++)	Pain (NRS, in acute group) Pain (NRS, in chronic group)			**-** **+**				Catastrophizing (PCCL per point increase) univariate analysis (Beta 1.0, CI 0.44–1.57 (positive = more pain reduction) for decrease in pain at 6 months in acute shoulder pain patients but not in chronic shoulder pain patients. In the multivariate analysis catastrophizing is a negative predictor (less decrease of pain) in the chronic shoulder pain patients (Beta-0.62, CI −1.03- (−0.20)) and was no longer included in the acute pain patients; 4DSQ N.S.
**Ryall et al. 2007** [47]	(++)	Pain (^a^ADI)		**–**			**–**	**–**	Belief that problem is likely to be causing difficulties in 3 months N.S.; Brief Symptom Inventory (BSI) >2points N.S.; Depression Scale (HADS)-D > 7 for continuing pain at 12 months, frequent continuing pain, unremitting pain N.S.; HADS-A > 7 continuing pain at 12 months, frequent continuing pain, unremitting pain N.S.
Sindhu et al. 2012 [48]	(+)	Shoulder function (Computerized Adaptive Test)				**+**			FABP-*P* > 16 high FAB: the improvement of function was greater in low fear avoidance groups after adjustment for 8 disease categories. No difference was found for arthropathies, fractures, sprains and strains, postsurgical conditions.
**Smedbråten et al, 2018** [49]	(++)	Pain (NRS) Function (PSFS)					**+** **-**		In final multiple regression model, emotional distress (HSCL-25) associated with more pain (Beta 1.06, 95% CI 0.44–1.68, *p* = 0.001). Other significant predictors: pain intensity before treatment, duration of pain > 12 months. Emotional distress univariate significant, not included in the multiple regression model. Significant predictors were higher pre-treatment disability, pain duration > 12 months, concomitant neck pain, and a lower level of education.
**Van der Windt et al. 2007** [50]	(++)	Perceived recovery (^a^ADI) Shoulder disability (SDQ)			**-** **-**	**-** **-**	**-** **-**		Perceived recovery was measured by Likert scale. Catastrophizing (PCCL score) > 40 adjusted OR 0.94 (95% CI 0.52–1.68) for persisting symptoms, OR 1.32 (CI 0.78–2.24) for < 30% disability reduction; FABQ-*P* > 75 (0–100) adjusted OR 1.08 (CI 0.63–1.85) for persisting symptoms, OR 1.12 (0.568–1.85) for disability reduction; Somatization, measured by 4DSQ > 30 adjusted OR 1.46 (CI 0.63–3.42) for persisting symptoms, OR 1.49 (CI 0.74–3.01) for disability reduction; Distress 4DSQ > 12 adjusted OR 0.71 (CI 0.42–1.19) for persisting symptoms, OR 0.76 (CI 0.48–1.23) for disability reduction.
**Wolfensberger et al., 2016** [51]	(++)	Shoulder disability (DASH) Pain (Patient Global Impression of Change)			**+** **+**	**-** **+**	**+** **+**	**+** **+**	In the multivariable analysis factors were combined: HADS-A, HADS-D, and Pain Catastrophizing Scale (PCS) were associated with more disability (DASH, Beta 0.64 (95% CI 0.25–1.03, *p* = 0.002). Also, less Patient Global Impression of change associated with combination of: HADS-D + A + PCS + TSK (Beta 0.93, 95% CI 0.87–0.99, *p* = 0.026).

Overall study quality, high (++), moderate (+), low (0)

+ Statistically significant relationship was found; − no statistically significant relationship was found

^a^ *ADI* Author defined instrument, *4DSQ* Four-Dimensional Symptom Questionnaire, *ASES* the American Shoulder and Elbow Surgeons’ Scale, *BPI* Brief Pain Inventory, *CBT* cognitive behavioural therapy approach, *DASH (and QuickDASH)* (Quick) Disability of the Arm, Shoulder and Hand Questionnaire, *EQ-5D* EuroQol- 5 Dimension, *FABQ* Fear Avoidance Beliefs Questionnaire (FABQ-P: physical activity subscale; FABQ-W, work subscale), *FLEX-SF* Flexilevel Scale of Shoulder Function, *GE* graded exercise, *HADS* Hospital Anxiety and Depression Scale, *HSCL-25* Hopkins Symptoms Checklist, *MCID* A minimal clinically important difference, *MODEMS* Musculoskeletal Outcomes Data Evaluation and Management System, *NPRS* Numeric Pain Rating Scale, *NRS* Numeric Rating Scale, *OSS* Oxford Shoulder Score, *PCCL* Pain Coping and Cognition List, *PCS* Pain Catastrophizing Scale, *PSFS* Patient Specific Functional Scale, *PT* physical therapy, *RCT* randomized controlled trial, *SDQ* Shoulder Disability Questionnaire, *SF-36* Short Form Survey, *SPADI* Shoulder Pain and Disability Index, *SST* Simple Shoulder Test, *TSK* Tampa Scale of Kinesiophobia, *UC* usual care, *UCLA Scale* The University of California at Los Angeles Shoulder Score, *VAS* Visual Analog Scale, *WORC* Western Ontario Rotator Cuff Index

**Table 4:** Predictive utility of psychological factors on the outcome after surgical treatment for shoulder complaints. Bold font indicates high quality studies based on the SIGN review

Authors	Quality	Outcome	Beliefs		Cognitive Style		Affect: Distress		Effect
			1.Self efficacy / Coping	2. Expectation of recovery	3. Catastrophizing	4. Avoidance Coping Style	5. Depression	6. Anxiety / Worry/ Fear	
Cho et al. 2015 [52]	(+)	Pain (VAS) Pain And function (UCLA) Pain and function (ASES)					**+** **+** **+**	**+** **+** **+**	Twelve months follow-up association in the multivariate linear regression analysis HADS-D with VAS − 0.073 (CI − 0.298 - 0.152), with UCLA score − 0.027 (− 0.565–0.511), ASES score − 0.235 (− 1.49–1.96). HADS-A with VAS 0.12 (− 0.05–0.28), UCLA − 0.09 (− 0.49–0.31), ASES − 0.62 (− 1.91–0.67).
Dambreville et al. 2007 [53]	(+)	Pain (VAS)					**+**	**–**	Preoperative depression (HADS) associated with pain at one month in a multivariate analysis (*p* = 0.03), not significant in postoperative pain; Anxiety (HADS) N.S.
**Dekker et al. 2016** [54]	(++)	Pain (VAS) Shoulder Pain and function (OSS)					**+** **+**		Preoperative depression score revealed a strong negative correlation between preoperative HADS score and 6-week OSS (*r =* − 0.490, *p* < .01), HADS and 6-month OSS (*r =* − 0.626, *p <* .01) and HADS and 6-month satisfaction (*r =* − 0.259, *p* < .05). There as strong positive correlation (*r =* − 0.508, *p* = 0.01) between HADS score and 6-month pain scores.
**George et al 2016** [56]	(++)	Pain (BPI) Shoulder disability (QuickDASH)			**+** **+**	**+** **-**	**+**	**+**	Additional analysis using the risk groups for George et al. 2015 and Simon et al. 2020 (included in this review). Strong statistical evidence was found for ADRB2 and depressive symptoms for postoperative course (pain and disability), and GCH1 and anxiety symptoms for 12-month pain-intensity outcome. Interactions involving inflammatory genes with strong statistical evidence for the 12-month postoperative course outcome were: two different IL6 single-nucleotide polymorphisms and pain catastrophizing, and IL6 and depressive symptoms; KCNS1 and kinesiophobia for preoperative pain intensity but not for postoperative pain.
George et al. 2008 [55]	(+)	Pain (BPI)			**+**	**–**			Postoperative pain measured by BPI > 4 points. Baseline PCS was associated with baseline pain, PCS baseline high score and low-COMT-phenotype the relative risk of high postoperative shoulder pain 6.8 (CI 2.8–16.7); Fear of pain or kinesiophobia were not associated with baseline pain or postoperative outcome (FPQ-III and TSK-11), however postoperative outcome was not systematically analysed.
**George et al. 2015** [57]	(++)	Recovery (^a^ADI)			**+**	**+**			Additional analysis using the risk groups for George et al. 2016 and Simon et al. 2020 (included in this review). Additional analysis using the risk groups identified in Simon 2020 (included in this review). Pain recovery was defined by: current pain intensity at VAS 0/10 and worst pain intensity 2/10. PCS, the catastrophizing high risk subgroup (combination of COMT and PCS score) were less likely to recover at 12 months (HR 0.51, P = 0.002); FABQ-score high risk subgroup (combination of COMT and FABQ-score) was less likely to recover at 12 months (HR 0.69, *p* = 0.043).
Henn et al. 2007 [59]	(+)	Pain (VAS) Shoulder function (SST) Shoulder disability (DASH) Physical and mental health (SF-36)		**+** **+** **+** **+**					Preoperative expectation regarding the treatment (MODEM questionnaire): 6 questions, mean score: expectations were a significant independent predictor of better postoperative outcome scores (VAS (Beta 9.91, *p* = 0.005), DASH (Beta 11.93, *p* = < 0.001), SF-36, SST (Beta 15.34, *p <* 0.001)) at 12 months; Workers compensation in the multivariate model significant for VAS (Beta − 12.88, *p* = 0.009), DASH (Beta −9.12, *p* = 0.011), SST (Beta − 1.33, *p* = 0.038), SF-36.
Jain et al. 2018 [60]	(++)	Pain and disability (SPADI)				**+**	**–**		Linear mixed prediction models incorporating a covariance structure using all available follow-up time points (3, 6, 12, and 18 months) for a given patient. Higher FABQ physical activity score predicted higher SPADI scores (worse shoulder pain and function), p for interaction =0.001. Mental Health Inventory (MHI-5, distress) N.S.
Koorevaar et al. 2018 [61]	(+)	Shoulder disability (DASH)					**–**		Additional analysis using the risk groups for Korevaar et al. 2016 (included in this review). Comparison of group 1 (≥1 psychological disorder before and 12 months after surgery *n* = 32) and group 2 (no psychological disorders, *n* = 110). DASH scores before (Group 1 55.5 [SD 19.8], Group 2 35.3 [SD 21.2], *p <* 0.001) and 12 months after shoulder surgery (Group 1 34.8 [SD 20.5], (Group 2 12.1 [SD 12.1], *p* < 0.001) were significantly higher in patients with symptoms of psychological disorders. Change of DASH score (*p* = 0.559) and MCID (% complete recovery, *p* = 0.284) were not different between the two groups. No adjustment for differences in baseline variables.
**Koorevaar et al. 2016** [62]	(++)	Shoulder disability (DASH)					**+**	**+**	Additional analysis using the risk groups for Korevaar et al. 2018 (included in this review). Preoperative 4DSQ (distress, depression, anxiety, and somatization) was adjusted for age, gender and preoperative DASH score, associated with less of an improvement in DASH score.
Lau et al. 2019 [63]	(+)	Pain and function (ASES)					**+**	**+**	Same patient samples as Lau et al.2019 (included in this review). A higher score of depression or anxiety related to the shoulder had a negative correlation with the postoperative (*r =* −0.31, *p* = 0.0001; and *r =* − 0.31, *p* = 0.0003, respectively) ASES scores, but a positive correlation (*r =* 0.50, *p <* 0.0001; and *r =* 0.43, *p* < 0.0001, respectively) with the change in ASES score (pre to post operatively). No multivariate analysis and no adjustment for other factors.
Lau et al. 2020 [64]	(+)	Pain and function (ASES)					**+**	**+**	Same patient samples as Lau et al.2019 (included in this review). Subjects were classified as those with diagnosed clinical depression/anxiety and those with symptoms but no diagnosis. Regardless classification, there was a strong association for depression between improvement in ASES scores and changes in shoulder-related depression (*r =* 0.68 [with clinical diagnosis], *r =* 0.75 [without clinical diagnosis]). Regarding anxiety, there was a moderate association between improvement in ASES scores and changes in shoulder-related depression (*r =* 0.56 [with clinical diagnosis], *r =* 0.74 [without clinical diagnosis]. No multivariate analysis and no adjustment for other factors.
**Oh et al.72012** [65]	(++)	Shoulder function (SST) Shoulder function (Improvement Constant-Murley score) Physical and mental health (SF-36)		**+** **+** **+**					Patients were classified into low (33%), middle (33%), and high (33%) expectation or concern groups (based on mean expectation (MODEMS score) or concern score). High-expectation group more improvement on SST (*p* = 0.24), Constant Murley scores (*P* < .001), and the SF-36 Physical Function (*P* = 0.006) compared to low expectation group. High-concern group no significant improvement compared with low-concern group on SST (*p* = 0.9), Constant Murley scores (*p* = 0.7), and SF-36 physical function (*p* = 0.4).
Potter et al. 2015 [66]	(+)	Pain (VAS) Shoulder function (SST) Pain and function (ASES)					**-** **-** **-**		Score stratified based on Distress Risk Assessment Method. No significant differences between group with preop distress and those non-distressed. VAS MCID in non-distressed group 59% and in distressed group 81% (OR, 2.91; 95% CI, 0.92–9.14; *p* = 0.06). SST MCID in non-distressed 89% and distressed 81% (OR, 0.54; 95% CI, 0.14–2.07; *p* = 0.36). ASES MCID in non-distressed 86% and distressed 88% (OR, 1.21; 95% CI, 0.28–5.32; *p* = 0.80).
Ravindra et al. 2018 [67]	(+)	Pain (VAS) Pain and function (ASES)					**-** **-**		Correlation coefficients were calculated for VAS and ASES at 1 year for the following independent variables: preoperative demographic factors, MRI tear characteristics. Correlation coefficients were calculated for preoperative VAS scores and ASES and WORC, SST, and SF-36 scores Significant correlation found for higher 1-year VAS scores and higher preoperative VAS pain scores, narcotic use, and low WORC scores (both composite and emotion). Correlation with higher ASES scores at 1-year was found for higher preoperative VAS scores and increased supraspinatus atrophy.
**Simon et al. 2020** [58]	(++)	Active shoulder range of motion (flexion and abduction) Movement evoked pain (NPRS)					**-** **+**	**-** **-**	Additional analysis using the risk groups identified in George et al. 2015 and 2016 (included in this review). There were no significant findings for psychological factors and active range of motion. Depressive symptoms were found to mediate the causal pathway in the high-risk subgroup for increased movement-evoked pain intensity at 12 months (*p* = 0.038). The mediation effect accounts for 53% of the total effect of the high-risk group on 12-month movement-evoked pain.
Thorpe et al. 2018 [68]	(+)	Pain and function (ASES)			**+**		**+**	**+**	After adjustment for gender, workers compensation status, alcohol use and confidence in surgical outcome, cluster with poor psychological health was independently associated with worse ASES score at all time points (regression coefficient for ASES: 3 months after surgery −15 [95% CI, −23 to −8], *p <* 0.001); and 12 months after surgery −9 [95% CI, − 17 to − 1], *p* = 0.023). ASES scores improved in both clusters from before surgery to 12 months after surgery equally (regression coefficient for ASES: cluster 2 31 [95% CI, 26–36], *p* < 0.001); cluster 1 31 [95% CI, 23–39], p < 0.001).
Valencia et al. 2014 [69]	(+)	Pain (BPI) Shoulder disability (DASH)			**-** **+**		**-** **+**		PCS no significant correlation with 6 months pain, significant correlation with DASH at 6 months (*r =* 0.225); PHQ 9 not significant for pain but significant for disability (DASH, *r =* 0.287).
Woollard et al. 2017 [70]	(+)	Disability (^a^ADI)				**+**			Criteria for functional disability postoperative: (1) Global rating of change ≥ + 5, (2) ≥17-point improvement on the WORC from baseline to 6-months postoperative. Logistic regression model including (1) surgery on dominant shoulder, (2) work compensation status, (3) modified job duty, (4) baseline FABQ-work, internal rotation strength. FABQ-Work was associated with a lower success rate (OR 0.92, 95% CI 0.85–1.00). FABQ work subscale of ≤25 and surgery on the dominant shoulder were both strongly predictive of being a responder to surgery (FABQ work ≤25 points Beta 2.73, OR 15.29 (95% CI 2.30–101.9), p = 0.005)
Yeoman et al. 2012 [71]	(+)	Pain (VAS) Shoulder pain and function (OSS)					**-** **-**	**-** **-**	HADS (> 7 points) no significant difference in the postoperative function and VAS in the depression versus the no depression group (6 weeks follow-up); HADS (> 7 points) no significant difference in the postoperative function and VAS in the anxiety versus the no anxiety group (6 weeks follow-up).

Overall study quality, high (++), moderate (+), low (0)

+ Statistically significant relationship was found; − no statistically significant relationship was found

^a^ *ADI* Author defined instrument, *4DSQ* Four-Dimensional Symptom Questionnaire, *ASES* the American Shoulder and Elbow Surgeons’ Scale, *BPI* Brief Pain Inventory, *CBT* cognitive behavioural therapy approach, *DASH (and quickDASH)* (Quick) Disability of the Arm, Shoulder and Hand Questionnaire, *EQ-5D* EuroQol- 5 Dimension, *FABQ* Fear Avoidance Beliefs Questionnaire (FABQ-P: physical activity subscale; FABQ-W, work subscale), *FLEX-SF* Flexilevel Scale of Shoulder Function, *GE* graded exercise, *HADS* Hospital Anxiety and Depression Scale, *HSCL-25* Hopkins Symptoms Checklist, *MCID* A minimal clinically important difference, *MODEMS* Musculoskeletal Outcomes Data Evaluation and Management System, *NPRS* Numeric Pain Rating Scale, *NRS* Numeric Rating Scale, *OSS* Oxford Shoulder Score, *PCCL* Pain Coping and Cognition List, *PCS* Pain Catastrophizing Scale, *PSFS* Patient Specific Functional Scale, *PT* physical therapy, *RCT* randomized controlled trial, *SDQ* Shoulder Disability Questionnaire, *SF-36* Short Form Survey, *SPADI* Shoulder Pain and Disability Index, *SST* Simple Shoulder Test, *TSK* Tampa Scale of Kinesiophobia, *UC* usual care, *UCLA Scale* The University of California at Los Angeles Shoulder Score, *VAS* Visual Analog Scale, *WORC* Western Ontario Rotator Cuff Index

### Clinical intervention and time from onset

#### Conservative intervention

Among the 20 studies on conservative intervention, four addressed patients with subacute MSD, five addressed patients with chronic MSD and 11 did not specify time from onset or presented a mixed population. All six MPF were investigated (Table 5).

**Table 5:** Classification of studies based on the relationship between modifiable psychological constructs and outcome. Relationship between constructs and outcome is further classified based on clinical intervention, time from onset, and quality of study (high/ moderate) in each cell. Bold font indicates high quality studies. The term predictive refers to statistically significant effects observed in the studies

	Predicting outcome	1.Self efficacy / Coping	2. Expectation of recovery	3. Catastrophizing	4. Avoidant Coping Style	5. Depression	6. Anxiety / Worry/ Fear
**Conservative**							
Number of studies that psychological factors found to:							
Predicting outcome		1	3	2	3	4	2
Not predicting outcome		1	3	3	3	8	5
Subacute	Yes				**High:** [44]		
	No			**High:** [50] Moderate: [42]	**High:** [50] Moderate: [42]	**High:** [50] Moderate: [39, 42]	Moderate: [42, 44]
Chronic	Yes		**High:** [43]	**High:** [51]	**High:** [51]	**High:** [51] Moderate: [38]	**High:** [37, 51]
	No	**High:** [36]				**High:** [36, 43]	Moderate: [38]
Not specified / mixed	Yes	**High:** [34]	**High:** [34, 45]	**High:** [46]	Moderate: [48]	**High:** [40, 49]	
	No		**High:** [35, 47] Moderate: [33]	Moderate: [41]	Moderate: [41]	**High:** [4, 35, 47]	**High:** [4, 47]
**Surgery**							
Number of studies that psychological factors found to:							
Predicting outcome			2	5	4	9	6
Not predicting outcome					1	6	3
Subacute	Yes				**High:** [60]		
	No					**High:** [60]	
Chronic	Yes		**High:** [65] Moderate: [59]			Moderate: [52, 63, 64]	Moderate: [52, 63, 64]
	No					**High**: [58]	**High:** [58]
Not specified / mixed	Yes			**High:** [56, 57] Moderate: [55, 68, 69]	**High:** [56, 57] Moderate: [70]	**High:** [54, 56, 62] Moderate: [53, 68, 69]	**High:** [56, 62] Moderate: [68]
	No				Moderate: [55]	Moderate: [61, 66, 67, 71]	Moderate: [53, 71]

#### Surgical intervention

Among the 20 studies on surgical intervention, one addressed patients with subacute MSD, five addressed patients with chronic MSD, and fourteen studies did not specify time from onset or presented a mixed population. Five of six MPF were addressed. There were no studies investigating the construct of self-efficacy for surgical cases, Table 5.

### Modifiable psychological domains and constructs

In this sample, the domains of “coping” and “affect” were most investigated, 14 (40%) publications and 29 (73%) publications respectively, and the domain of “beliefs” was least investigated, 9 (23%) publications (Tables 3, 4 and 5). Of the six predefined constructs, depression (Domain: Affect) was the most studied construct, 27 (68%) publications, and self-efficacy (Domain: Beliefs), the least studied, two publication (5%). For surgical care, we found evidence for catastrophizing, avoidant coping, depression, anxiety, and expectation of recovery as predictors of outcome. In patients undergoing conservative intervention the evidence was either against (catastrophizing, depression, anxiety) or inconclusive (self-efficacy, expectation of recovery, avoidant coping) for the predictive value of psychological factors on outcome. The following provides details of the prognostic value of each MPF in patients with shoulder problems managed conservatively or surgically.

### Domain: coping

#### Catastrophizing

Catastrophizing as a predictor of outcome was explored in ten publications (five surgical, five conservative). In seven publications (five (100%) surgical [two high quality], two (40%) conservative [two high quality]) catastrophizing predicted at least one outcome. Therefore, based on this review, catastrophizing in surgical cases fell into Category 1, evidence for, while for conservative cases it was Category 3, evidence against.

#### Avoidant coping/fear avoidance

Avoidant coping as a predictor of outcome was explored in eleven publications (five surgical, six conservative). In seven publications (four (80%) surgical [three high quality], three (50%) conservative [two high quality]) avoidant coping/fear avoidance predicted at least one outcome. Therefore, based on this review, avoidant coping/fear avoidance in surgical cases fell into Category 1, evidence for, while for conservative cases it was Category 2, inconclusive.

### Domain: affect

#### Depression

Depression as a predictor of outcome was explored in 27 publications (15 surgical, 12 conservative). In 14 publications (nine (60%) surgical [four high quality], four (33%) conservative [three high quality]) depression predicted at least one outcome. Therefore, based on this review, evidence for depression in surgical cases fell into Category 1, evidence for, while for conservative cases it was Category 3, evidence against.

#### Anxiety

Anxiety as a predictor of outcome was explored in 16 publications (nine surgical, seven conservative). In eight publications (six (67%) surgical [three high quality], two (29%) conservative [two high quality]) anxiety predicted at least one outcome. Therefore, based on this review, evidence for anxiety as a predictor in surgical cases fell into Category 1, evidence for, while for conservative cases it was Category 3, evidence against.

### Domain: beliefs

#### Self-efficacy

Self-efficacy as a predictor of outcome was explored in two publications (two conservative [two high quality]). In one publication (50%) self-efficacy predicted at least one outcome. Therefore, based on this review, evidence for self-efficacy as a predictor in conservative cases fell into Category 2, inconclusive evidence.

#### Expectation of recovery

Expectation of recovery as a predictor of outcome was explored in eight publications (two surgical, six conservative). In five publications (two (100%) surgical [one high quality], three (50%) conservatives [three high quality]) expectation of recovery predicted at least one outcome. Therefore, based on this review, evidence for expectation of recovery as a predictor in surgical cases fell into Category 1, evidence for, while for conservative cases it was Category 2, inconclusive evidence.

## Discussion

In this study we explored the relationship between MPF and outcomes in patients with shoulder disorders, within the context of management (conservative, surgical) and temporal framework (time from onset). The main finding of this review is that psychological factors affect recovery in patients with shoulder pain managed surgically. However, MPF was not associated with outcome in patients receiving conservative care for shoulder disorders, regardless of duration of pain. This suggests that type of clinical management and time from onset are critically important variables to consider when defining the prognostic value of MPF on outcome in patients with MSD.

### Previous systematic reviews

Previous systematic reviews [11, 18, 22] have explored the association between MPF and outcome in patients with shoulder conditions including those receiving conservative and surgical care [18, 25], conservatively managed patients only [19, 20], patients with selective diagnoses, [21, 25–28], patients undergoing arthroplasty [29] or with conditions associated with chronic shoulder pain [11, 25]. However, they did not account for confounding factors that may impact this relationship, such as the approach to management (conservative, surgical) and time from onset. In addition, these reviews explored this topic through a narrow lens considering only several psychological factors or specific diagnoses. Therefore, previous reviews provide a limited perspective on the relationship between MPF and patients with shoulder conditions. The question that was addressed in this review was broad and included all reported diagnoses, time from onset, approaches to management, and did not predefine MPF or outcome.

We classified studies based on conservative and surgical intervention and all diagnostic phases from acute through chronic. Furthermore, we did not predefine psychological factors or outcomes but rather extracted from the reviewed studies. In addition, we applied no language or publication timeframe restrictions in our search allowing for a broad body of literature from which this topic could be explored. Defining and focusing specifically on psychological factors that are modifiable is relevant as these factors are responsive to short-term intervention, as opposed to more refractory psychiatric diagnoses that are more difficult to manage [72]. For these reasons, the findings of this review may be clinically relevant in that they may guide the approach to preoperative care.

### Evidence supporting MPF

In this review six distinct MPF were identified. However, for most of these factors, few studies have explored their relationship with outcome and not all were graded as high quality. For the purpose of this review, our conclusion regarding the effect of each factor on outcome was based on the preponderance of the included references. However, it should be noted that very small numbers of studies or nearly equivocal numbers of studies supporting or refuting the findings were used to determine our conclusions. This was particularly true in the review of those studies on conservatively managed cases.

This can be highlighted by examining the findings for individual MPF. Depression was the most widely studied construct with 27 studies (12 conservative and 15 surgical) management. In the case of conservative management, four predicted outcome and eight did not, a clear conclusion. In the case of surgical management, nine predicted and six did not, also a clear finding. In contrast, self-efficacy was far less studied with only two studies for conservatively managed cases and none for surgical. In one study self-efficacy predicted outcome and in one it did not, and therefore the conclusion must be weighed carefully. Therefore, it is important to consider the total number of studies reviewed when interpreting the relationships between each individual MPF and outcome (Table 5).

### Approach to management PPROACH TO MANAGEMENT

The implications of this review suggest that MPF are important considerations for those patients with MSD who are managed surgically. Our findings show that there is evidence for the predictive value of expectation of recovery, catastrophizing, avoidant coping, depression, and anxiety in patients receiving surgically intervention. In this group, there was no evidence that self-efficacy affected outcome. The results suggest the importance of assessment of these MPF as a part of routine surgical care for patient with shoulder disorders. In contrast, for those patients managed conservatively, the evidence for self-efficacy, expectation of recovery, and avoidant coping was equivocal and requires further study. However, there was no evidence for catastrophizing, depression and anxiety affecting outcome in this group. When evaluating the findings for each construct as it relates to management it is important to consider not only the number of studies but also the quality of studies informing the conclusion, as more high-quality studies were noted for conservative management (Table 5).

### Time from onset

In this review we explored the temporal influence, represented as time from onset, on the relationship between MPF and outcome. Time from onset of shoulder pain was not defined in 60% of the included references (14 of the surgical studies [82%] and ten of the conservative studies [50%]). When interpreting the findings, it is important to recognize that typically surgical intervention occurs during the chronic phase, after failed conservative management often recommended during earlier phases [73]. Therefore, it may be reasonable to conclude that in the absence of trauma, the majority of patients undergoing surgical intervention were likely in the chronic phase [74]. Although less than 20% of the surgical studies reported time from onset, in those that did, a relationship between MPF and outcome was found.

In the case of conservative intervention, it is difficult to draw conclusions regarding the temporal impact of MPF on outcome. This is because among those studies that did report time from onset, the findings were either inconclusive or against the predictive value of MPF on outcome. Therefore, we believe time from onset deserves further study in this group.

### Limitations

Many included studies were small and may therefore not have sufficient power to capture a clinically relevant influence of the subgroups we have defined for this review. None of the included studies investigated all predefined constructs and therefore the full impact of these variables cannot be completely described. In addition, not all MPF were equally explored. Furthermore, some psychological constructs are complex, such as catastrophizing, which may be considered a belief or a coping strategy. For example, two studies that used the Pain Coping Scale designated catastrophizing as a coping strategy [42, 50]. Yet most studies used the Pain Coping Scale to assess the impact of beliefs on expectation of outcome [46, 55–57, 69]. There is no gold standard for the definition and classification psychological constructs. In this review, catastrophizing was assigned to the coping domain based on the opinion of a clinical mental health expert. However, future studies need to clarify the difference between beliefs and coping strategies and their impact on treatment outcome. In one study, the Orebro, a composite measure for MPF and other variables associated with outcome, was used to assess MPF [37]. Due to its composite nature, it was not possible to include the findings for specific MPF in this review. However, composite instruments may allow for the assessment of several domains simultaneously and may have clinical utility, compared to the methods in this review that explored each MPF individually. The impact of treatment for the MPF (i.e., medication, psychological interventions) on shoulder outcomes was not addressed in this review. The limitations of our review reflect the lack of a strong literature base, including the heterogeneity of study populations, which precluded the possibility of a meta-analysis [22, 25]. Future studies need to address these methodological shortcomings.

### Future studies

There is mention of the importance of assessing psychological factors in clinical practice guidelines for managing shoulder pain [6]. However, this does not seem to be a routine part of clinical practice as is apparent from the limited number of studies found for this review. To gain deeper insight into how to explore the role of psychological factors as predictors of outcome, it is informative to look to the spine literature. Compared to the management of shoulder disorders, an extensive literature base drives clinical management of psychological factors associated with low back pain. Consistent evidence supports the role of these psychological factors on prognosis [17] and the relationship with outcome for patients with low back pain [75, 76]. However, there are limitations in generalizing the findings to other musculoskeletal disorders such as shoulder pain. While the overall relationship of low back pain with physical functioning and MPF has been described, it is unclear if the same relationship may exist for other musculoskeletal conditions.

One consideration is the relationship between psychological factors and the natural history/tissue healing associated with various musculoskeletal conditions. For instance, in patients with low back pain, fear of pain is a strong predictor of outcome [75, 76]. The concept that pain does not equal damage, an important message to patients with spinal pain, may not be relevant for patients with shoulder conditions. Furthermore, while studies on back pain may inform the methodologies and research questions for shoulder pain populations, researchers must be prudent in recognizing the limitations of transposing these ideas. For example, many of the tools used to measure psychological constructs have not been validated for shoulder complaints [20]. Finally, other psychological responses to pain, such as anger, have been studied in other musculoskeletal conditions, yet are not addressed in the shoulder literature [77]. Future studies should focus on developing shoulder-specific instruments, clinical management, time from onset and all relevant psychological factors that are potentially modifiable as they relate to outcome.

## Conclusions

Based on this review, expectation of recovery, catastrophizing, avoidant coping style, depression, and anxiety were the MPF most predictive of outcome in surgically managed patients with shoulder complaints. This provides sufficient evidence to suggest that implementing a biopsychosocial care paradigm to this population may be advantageous. In patients undergoing conservative intervention the evidence was either against (catastrophizing, depression, anxiety) or inconclusive (self-efficacy, expectation of recovery, avoidant coping) for the predictive value of psychological factors on outcome. However, future high-quality comparative investigations and those assessing understudied constructs may shed more light on the prognostic value of MPF on outcome in this population. There is clearly a place for the study of psychological factors associated with shoulder disorders. Further investigation of all psychological factors may provide deeper insight into understanding patients with shoulder MSD, and best approaches to clinical management. 

## Data Availability

Not applicable. All data are available in public domains.

## Appendix 1

### Search strategies

1 A: Embase Search July 3, 2019.

Elsevier© 2019 RELX Intellectual Properties SA.

Part B. Medline July 3, 2019.

Ebsco Host.

### Appendix 2

SIGN Quality Assessment. Bold font indicates high quality studies based on the SIGN review.

-, low quality, few or no criteria fulfilled.

Columns 1–14 in Appendix 2A are in response to the following questions:

1. Study question focused? 2. Included groups selected from source population that are comparable. 3. The study indicate how many who were asked to take part did so. 4. The likelihood that some eligible subjects might have the outcome at the time of the enrolment is assessed and taken into consideration. 5. What are the percentage of individuals recruited that dropped out before the study was completed. 6. Comparison is made between full participants and those lost to follow-up. 7. Outcomes clearly defined. 8. The assessment of outcome is made blind to exposure status. 9. Where blinding was not possible, there is some recognition that knowledge of the exposure status could have influenced the assessment of outcome. 10. The method of assessment of exposure is reliable? 11. Evidence from other sources is used to demonstrate that the method of outcome assessment is valid and reliable. 12. Exposure or prognostic factor assessed more than once? 13. Main potential confounders identified and taken into account in analysis. 14. Have confidence intervals been provided. 15. Overall assessment of risk of bias (++/+/−/0).

2 B: Randomized clinical trials.

Columns 1–10 reflect the SIGN questions listed in the legend below the Appendix.

-, low quality, few or no criteria fulfilled.

Columns 1–10 in Appendix 2B are in response to the following questions:

1. Clearly and focused question. 2. The assignment of subjects to treatment groups are randomized? 3. An adequate concealment method is used? 4. The design keeps subjects and investigators ‘blind’ about treatment allocation? 5. The groups are similar at start of the trials? 6. The only difference between the groups is the treatment under investigation? 7. All relevant outcomes are measured in a standard, valid and reliable way? 8. What percentage of the subjects recruited into each treatment arm dropped out before the study was completed? 9. All the subjects are analysed in the groups to which they were allocated (Intention to treat analysis)? 10. Where the study is carried out at more than one site, results are comparable for all sites? Overall quality of the study? (++/+/−/0).

**Table:**

#	Query	Results 07/2019
1	‘shoulder pain’/exp. OR ‘rotator cuff injury’/exp. OR ‘bursitis’/exp	28,132
2	‘shoulder’/exp. AND ‘pain’/exp	12,131
3	shoulder NEAR/5 (bursitis OR ‘adhesive capsulitis’ OR periarthriti* OR frozen OR impinge* OR tend*nitis OR pain*)	26,797
4	‘rotator cuff’ NEAR/5 (bursitis OR ‘adhesive capsulitis’ OR periarthriti* OR frozen OR impinge* OR tend*nitis OR pain*)	3309
5	#1 OR #2 OR #3 OR #4	43,049
6	‘anxiety’/mj OR ‘anxiety disorder’/mj OR ‘catastrophizing’/mj OR ‘panic’/mj OR ‘coping behavior’/mj OR ‘adaptive behavior’/exp./mj OR ‘illness behavior’/exp./mj OR ‘attitude to illness’/exp./mj OR ‘self concept’/exp./mj	181,113
7	(fear NEAR/3 avoid*):ab,ti OR ((psychosocial OR psychological OR ‘bio psychological’) NEAR/3 (factor* OR model*)):ab,ti OR (coping NEAR/3 (behavi*r OR skill* OR pain OR strateg* OR ability)):ab,ti OR (pain NEAR/3 (catastrophizer* OR catastrophiser*)):ab,ti OR (catastrophic NEAR/3 (thinking OR thought*)):ab,ti OR catastrophizing:ab,ti OR catastrophising:ab,ti OR catastrophization:ab,ti OR catastrophisation:ab,ti OR kinesiophobia:ab,ti	70,556
8	#6 OR #7	238,308
9	#5 AND #8	580

**Table:**

#	Query	07/2019
1	(MH “Shoulder Pain”) OR (MH “Shoulder Impingement Syndrome”) OR (MH “Rotator Cuff”) OR (MH “Bursitis+”)	14,984
2	(MH “Shoulder Joint”) AND (MH “Pain+”)	2307
3	AB (shoulder N5 (bursitis OR “adhesive capsulitis” OR periarthriti* OR frozen OR impinge* OR tend#nitis OR pain*)) OR TI (shoulder N5 (bursitis OR “adhesive capsulitis” OR periarthriti* OR frozen OR impinge* OR tend#nitis OR pain*))	13,544
4	AB (“rotator cuff” N5 (bursitis OR “adhesive capsulitis” OR periarthriti* OR frozen OR impinge* OR tend#nitis OR pain*)) OR TI (“rotator cuff” N5 (bursitis OR “adhesive capsulitis” OR periarthriti* OR frozen OR impinge* OR tend#nitis OR pain*))	1525
5	S1 OR S2 OR S3 OR S4	24,155
6	(MH “Anxiety”) OR (MH “Anxiety Disorders”) OR (MH “Catastrophization”) OR (MH “Fear”) OR (MH “Panic”) OR (MH “Adaptation, Psychological+”) OR (MH “Illness Behavior”) OR (MH “Self Efficacy”)	256,353
7	TI (Fear N3 avoid*) OR TI ((Psychosocial OR psychological OR bio-psychological) N3 (factor* OR model*)) OR TI (Coping N3 (behavi#r or skill* OR pain OR strateg* OR ability)) OR TI ((pain N3 (catastrophizer* or catastrophiser*))) OR TI ((catastrophic N3 (thinking or thought*))) OR TI ((catastrophizing or catastrophising or catastrophization or catastrophisation)) OR TI kinesiophobia OR AB (Fear N3 avoid*) OR AB ((Psychosocial OR psychological OR bio-psychological) N3 (factor* OR model*)) OR AB (Coping N3 (behavi#r or skill* OR pain OR strateg* OR ability)) OR AB ((pain N3 (catastrophizer* or catastrophiser*))) OR AB ((catastrophic N3 (thinking or thought*))) OR AB ((catastrophizing or catastrophising or catastrophization or catastrophisation)) OR AB kinesiophobia	55,863
8	S6 OR S7	293,180
9	S8 AND S5	430

**Table:**

2 A: Cohort studies Columns 1–14 reflect the SIGN questions listed in the legend below the Appendix.																
Author and year	1	2	3	4	5	6	7	8	9	10	11	12	13	14	Overall Quality	
**Chester et al. 2016, Chester et al. 2019**	Y	NA	Y	NA	25%	Y	Y	Y	NA	Y	Y	N	Y	Y	++	**9**
Cho et al. 2015	Y	NA	Y	NA	19%	N	Y	?	N	Y	Y	N	?	Y	+	6
Dambreville et al. 2007	Y	NA	N	NA	0%	NA	Y	?	N	Y	Y	N	Y	N	+	5
**Dekker et al. 2016**	Y	Y	Y	NA	28%	N	Y	?	NA	Y	Y	N	Y	Y	++	**8**
**Engebretsen et al. 2010**	Y	Y	Y	NA	10%	Y	Y	Y	NA	Y	Y	N	?	Y	++	**8**
**Engebretsen et al. 2020**	Y	Y	N	NA	29%	N	Y	Y	NA	Y	Y	N	Y	Y	++	**8**
George et al. 2008	Y	NA	N	NA	19%	N	Y	?	?	Y	Y	N	?	N	+	4
**George et al. 2015, 2016, Simon et al. 2020**	Y	NA	Y	Y	25%	N	Y	Y	?	Y	Y	N	Y	Y	++	**9**
Henn et al. 2007	Y	?	N	NA	0%	NA	Y	NA	?	Y	Y	N	N	N	+	3
**Jain et al 2018**	Y	Y	Y	NA	30%	N	Y	Y	NA	Y	Y	N	N	Y	++	**8**
**Karel et al 2017**	Y	Y	Y	NA	30%	Y	Y	Y	NA	Y	Y	N	Y	Y	++	**10**
**Kennedy et al 2006**	Y	Y	N	NA	0%	NA	Y	Y	Y	Y	Y	N	N	Y	++	**8**
**Koorevaar et al 2016**	Y	Y	Y	NA	9%	Y	Y	Y	NA	Y	Y	N	Y	Y	++	**10**
Koorevaar et al. 2018	Y	Y	Y	NA	22%	N	Y	Y	NA	Y	Y	N	NA	N	+	7
Kromer T.O. 2014	Y	Y	Y	NA	2%	N	Y	NA	NA	Y	Y	N	?	Y	+	7
Kuijpers et al. 2006	Y	NA	N	NA	8%	Y	Y	Y	NA	Y	Y	N	Y	Y	+	7
**Laslett et al. 2015**	Y	Y	Y	N	16%	Y	Y	?	?	Y	Y	N	?	Y	++	**8**
Lau et al. 2019; Lau et al. 2020	Y	Y	Y	NA	9%	N	Y	N	N	Y	Y	N	N	N	+	6
**O’Malley et al, 2004**	Y	Y	N	NA	39%	Y	Y	Y	NA	Y	Y	N	Y	N	++	**8**
**Oh et al 2012**	Y	Y	Y	NA	26%	N	Y	Y	NA	Y	Y	N	?	Y	++	8
Potter et al. 2015	Y	?	Y	NA	18%	N	Y	Y	NA	Y	Y	N	NA	Y	+	7
Ravindra et al. 2018	Y	Y	N	NA	22%	N	Y	?	N	Y	Y	N	N	N	+	5
**Reilingh et al. 2008**	Y	Y	Y	NA	20–8%	Y	Y	?	Y	Y	Y	N	Y	Y	++	**9**
**Ryall et al. 2007**	Y	Y	Y	NA	16.5%	Y	Y	?	N	Y	Y	N	Y	Y	++	**9**
Sindhu et al. 2012	Y	Y	N	NA	57–47%	N	Y	NA	?	Y	Y	N	?	N	+	5
**Smedbråten et al. 2018**	Y	Y	Y	NA	31%	Y	Y	Y	N	Y	Y	N	Y	Y	++	**10**
Thorpe et al. 2018	Y	Y	Y	NA	10%	N	Y	?	N	Y	Y	N	Y	N	+	7
Valencia et al. 2014	Y	NA	N	Y	6.5%	N	Y	?	?	Y	Y	Y	Y	N	+	7
**Van der Windt et al. 2007**	Y	NA	N	NA	12%	Y	Y	Y	NA	Y	Y	N	Y	Y	++	**8**
**Wolfensberger et al., 2016**	Y	NA	Y	N	45%	Y	Y	Y	NA	Y	Y	N	Y	Y	++	**9**
Woollard et al. 2017	Y	Y	N	NA	25%	N	Y	?	N	Y	Y	N	Y	Y	+	7
Yeoman et al. 2012	Y	NA	N	NA	0%	NA	Y	NA	Y	Y	Y	Y	?	N	+	5

Y = yes; *N =* no;? = unclear; NA = not applicable.

++, high quality, most (≥60%) of the criteria fulfilled (if < 60% fulfilled, the conclusions of the study are very unlikely to alter the findings)

+, moderate quality: some criteria fulfilled (< 60%)

**Table:**

Author and year	1	2	3	4	5	6	7	8	9	10	Overall Quality	
Berk et al. 1977	Y	Y	?	?	?	Y	Y	NA	Y	NA	+	5
**Ekeberg et al 2010**	Y	Y	Y	Y	Y	Y	Y	1.8%	Y	NA	++	**8**
Geraets et al. 2005	Y	Y	Y	N	N	Y	?	16%	N	?	+	4
**Kvalvaag et al 2018**	Y	Y	Y	Y	Y	Y	Y	10%	Y	NA	**++**	**8**

Y = yes; *N =* no;? = unclear; NA = not applicable.

++, high quality, most (≥60%) of the criteria fulfilled (if < 60% fulfilled, the conclusions of the study are very unlikely to alter the findings)

+, moderate quality: some criteria fulfilled (< 60%)

## Abbreviations

DASH: Disabilities of the Arm, Shoulder and Hand
NPRS: Numeric Pain Rating Scale
NRS: Numeric Rating Scale
MPF: Modifiable psychological factors
MSD: Musculoskeletal shoulder disorders
PRISMA: Preferred Reporting Items for Systematic Reviews and Meta-analyses
RCT: Randomized controlled trials
SIGN: Scottish Intercollegiate Guidelines Network
SPADI: Shoulder Pain and Disability Index
VAS: Visual Analog Scale
